# A narrative review on the application of artificial intelligence in renal ultrasound

**DOI:** 10.3389/fonc.2023.1252630

**Published:** 2024-03-01

**Authors:** Tong Xu, Xian-Ya Zhang, Na Yang, Fan Jiang, Gong-Quan Chen, Xiao-Fang Pan, Yue-Xiang Peng, Xin-Wu Cui

**Affiliations:** ^1^ Department of Medical Ultrasound, Tongji Hospital, Tongji Medical College, Huazhong University of Science and Technology, Wuhan, China; ^2^ Department of Ultrasound, Affiliated Hospital of Jilin Medical College, Jilin, China; ^3^ Department of Medical Ultrasound, The Second Hospital of Anhui Medical University, Hefei, China; ^4^ Department of Medical Ultrasound, Minda Hospital of Hubei Minzu University, Enshi, China; ^5^ Health Medical Department, Dalian Municipal Central Hospital, Dalian, China; ^6^ Department of Ultrasound, Wuhan Third Hospital, Tongren Hospital of Wuhan University, Wuhan, China

**Keywords:** artificial intelligence, ultrasound, kidney, machine learning, deep learning

## Abstract

Kidney disease is a serious public health problem and various kidney diseases could progress to end-stage renal disease. The many complications of end-stage renal disease. have a significant impact on the physical and mental health of patients. Ultrasound can be the test of choice for evaluating the kidney and perirenal tissue as it is real-time, available and non-radioactive. To overcome substantial interobserver variability in renal ultrasound interpretation, artificial intelligence (AI) has the potential to be a new method to help radiologists make clinical decisions. This review introduces the applications of AI in renal ultrasound, including automatic segmentation of the kidney, measurement of the renal volume, prediction of the kidney function, diagnosis of the kidney diseases. The advantages and disadvantages of the applications will also be presented clinicians to conduct research. Additionally, the challenges and future perspectives of AI are discussed.

## 1 Introduction

Kidney disease is a serious public health problem affecting more than 10% of the global population (1). End-stage renal disease, which develops from various kidney conditions, eventually forces patients to finally rely on renal replacement treatment to prolong their lives (2). The many complications of end-stage renal disease have a significant impact on the physical and mental health of patients. Early and accurate diagnosis is therefore essential to slow the progression of kidney disease and improve the quality of life of people with kidney disease.

Over the past few decades, the early detection, diagnosis, and treatment of diseases have benefited greatly from the application of several medical imaging modalities, such as computed tomography (CT), magnetic resonance imaging (MRI), ultrasound, and X-ray (3). Ultrasound is often the test of choice for evaluating the kidney and perirenal tissue because it is real-time, accessible, and non-radioactive. However, in clinical practice, the interpretation and analysis of medical images have mostly been performed by specialized physicians, which has been highly dependent on long-term training and often prone to subjective judgments. Furthermore, due to the high subjective heterogeneity in visual interpretation, it is challenging to translate experience-based prediction into standardized practice (4).

In recent decades, with the rapid development of AI technology, there have been a large number of applications of AI in medical imaging that benefits clinicians a lot (5). It is widely used in disease diagnosis that rely on medical imaging, such as breast cancer (6), liver diseases (7), pancreatic cancer (8), thyroid nodules (9) and urology diseases (10). AI has been explored as a new potential tool to assist radiologists in clinical decision making to overcome the substantial interobserver variability in image acquisition and interpretation.

**Figure 1:**
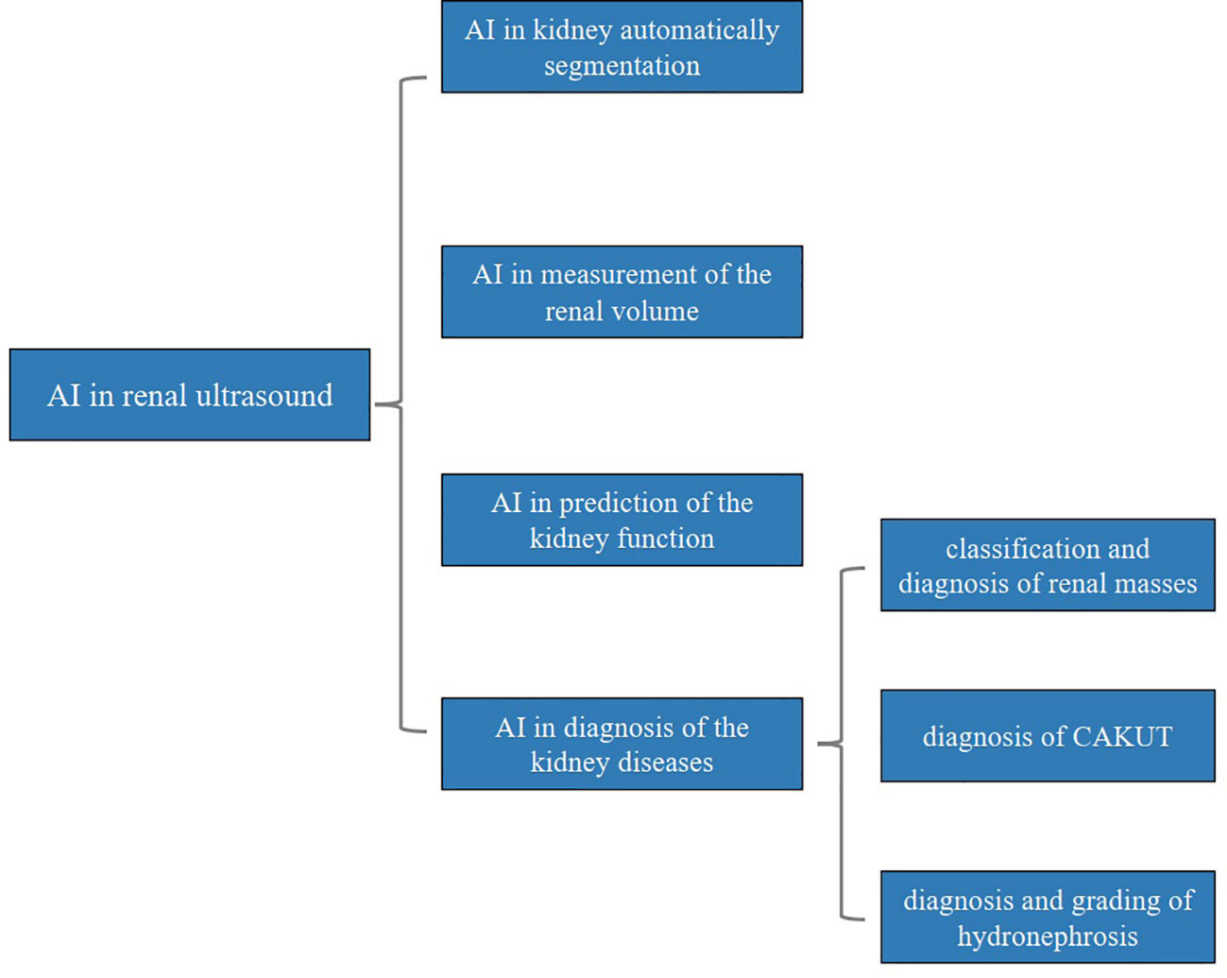
Main structure of this review. AI, artificial intelligence; CAKUT, congenital abnormalities of the kidney and urinary tract.

In this review, we comprehensively introduce the applications of AI in renal ultrasound, including automatic segmentation of the kidney, measurement of the renal volume, prediction of the kidney function, diagnosis of the kidney diseases. The advantages and disadvantages of the applications will also be presented to clinicians for research. Additionally, the challenges and future perspectives of AI in renal ultrasound are also discussed. The main structure of this review was presented in **Figure 1**.

## 2 Methods

We searched the PubMed and Web of Science databases for all research published in English up to 1 Jan 2023.In the search strategy, we used the search terms including “artificial intelligence”, “ultrasound”, and “renal”. The complete search strategy was available from the authors. We also included some narrative and systematic reviews to provide our readers with adequate details within the allowed number of references. In addition to the database searching, a hand search was performed, consisting of the reference lists of related articles and reviews and Google scholar search engines.

We mainly focused on AI in renal ultrasound images segmentation and the diagnostic and predictive capabilities of AI-assisted ultrasound in renal diseases. Hence, the articles were reviewed to determine the relevance based on the following criteria: studies that involved the application of AI methods to analyze ultrasound images of normal kidneys or renal diseases. The articles that were beyond this coverage were excluded.

All the titles and abstracts for all eligible articles were reviewed by two authors independently. If the abstracts were not relevant, then they were discarded, and the full-text articles were accessed. Further reviewing the full-text papers may lead to deserting some irrelevant documents and finally retaining articles that met the inclusion criteria in this review. When there was any discrepancy in the ultimately included articles, a consensus negotiation was reached to form the final inclusion result among the two authors.

## 3 Brief overview of artificial intelligence

AI was originally proposed at the Dartmouth Conference in 1956. The main goal of AI is to enable the performance of complex tasks that require human intelligence. The advancement of network technology has hastened the growth of AI innovation research, and significantly enhanced the technology’s usage. As an important component of the fourth industrial revolution, AI has changed dramatically our world, from facial recognition to smart homes. Particularly, AI methods have been found myriad applications in the medical image analysis field, driving it forward at a rapid pace, such as detection, segmentation, diagnosis, as well as risk assessment (5).

### 3.1 Machine learning

ML, a subfield of AI, is a general term for a class of algorithms. The basic idea of ML is to abstract a real-world problem into a mathematical model, to solve the model using mathematical methods to deal with the problem, and to evaluate whether and how well it has addressed the problem (11). Therefore, the process of ML could be simply summarized into three processes: training, validation, and testing.

According to the training methods, ML could be roughly divided into three types: supervised learning, unsupervised learning and reinforcement learning. Among them, supervised learning is the most widely used one in the field of medical imaging technology (12). It uses known input-outputs to train a model to map input data to output results, resulting in a function of inference, which is then able to infer output results from new input data. It is thus useful for classification, characterization and regression of the similarity between instances of similar results labels (12).

Consequently, in ultrasound imaging applications, expert-designed or radiomics-extracted image features can be used as input data to provide output predictions regarding subsequent disease outcomes. Commonly used ML techniques include linear regression, K-means, decision trees, random forests, support vector machines (SVMs), and neural networks (6).

### 3.2 Deep learning

**Figure 2:**
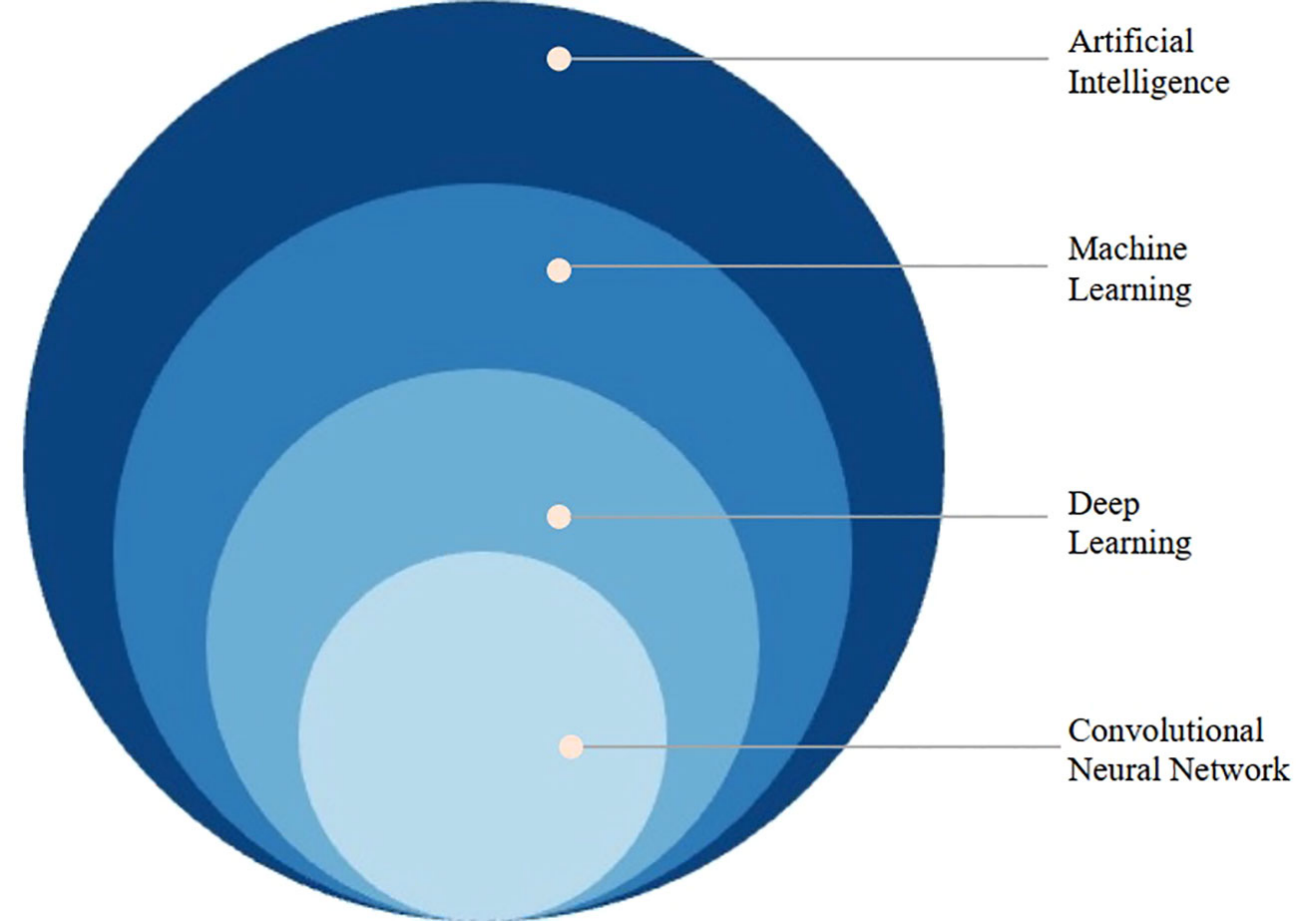
The Venn diagram of the artificial intelligence hierarchic terminology.

The concept of DL is derived from artificial neural networks, which is the most significant branch of ML, as illustrated in **Figure 2**. DL methods are representation-learning methods with multiple levels of representation, obtained by composing simple but non-linear modules that each transform the representation at one level (starting with the raw input) into a representation at a higher, slightly more abstract level (13). In contrast to traditional ML, which relies on manual feature extraction, DL is based on automatic feature extraction by machines. DL is highly dependent on data, and the larger the amount of data, the better its performance. Thus it has even surpassed human performance in tasks such as image recognition and facial recognition (13).

Currently, convolutional neural networks (CNNs) are the most popular type of DL architecture in the medical image analysis field (14). A typical CNN architecture consists of three parts: convolutional layers, pooling layers, and fully-connected layers. Generally, a CNN model has many convolutional layers and pooling layers. The convolutional layer and the pooling layer are alternately set (7). The convolution layer utilizes filters (convolution kernels) to filter regions of the image to extract the features. Pooling layers can reduce the dimensionality of data more effectively than convolutional layers, which can not only greatly reduce the amount of operations, but effectively avoid overfitting as well. The data processed by the convolutional and pooling layers are fed to the fully-connected layer. The fully connected layer can integrate local information with class discrimination (7).

The emergence and continuous development of DL has shed new light on medical image analysis and had a significant impact on both clinical applications and scientific research (15).

## 4 Application of AI in renal ultrasound

### 4.1 Automatic segmentation of the kidney

Kidney segmentation is the key and fundamental step for medical image analysis. The main motivations for kidney segmentation in clinical practice are: (a) Evaluation of kidney parameters, namely its size and volume, to diagnose potential diseases; (b) Assessment of renal morphology and function; (c) Localization of abnormalities or pathologies present in the kidney; (d) Facilitating the decision-making process, helping in the treatment/interventional planning; (e) Post-operative follow-up after a renal intervention (16). In the current clinical work, there are three main types of methods used in studies on kidney segmentation: manual, semi-automatic and fully automatic. Kidney image segmentation that is performed manually or semi-automatically relies heavily on timing-consuming manual tasks that also lead to higher inter-operator variability (17). Thus, automatic segmentation methods for kidney ultrasound image using AI are proposed, as summarized in **Table 1**.

**Table 1:** Summary of the application of AI in the renal ultrasound for automatic segmentation.

Task	Algorithms	Data source	Size	Results	Ref.
Automatic Segmentation	A framework combining with nonlocal total variation image denoising, distance regularized level set evolution and shape prior	synthetic and real ultrasound images of left kidney	/	Sen=95% Spe=96%	(18)
	Based on DeepLap and VGG16 CNNs incorporating transfer learning network, boundary distance regression network and kidney pixel classification	sagittal view of the kidney in children	289 images	Sen=94% Dice=94%	(19)
	A deep neural network consists of a multi-scale feature pyramid, a multi-branch encoder and a master decoder	clinical medical records of two centers	500 images	Spe=99.5%	(20)

CNN, convolutional neural network; Sen, sensitivity; Spe, specificity; Dice, Dice coefficient.

/, not mentioned.

Based on 2D ultrasound, Yang et al. developed a framework which was combined with nonlocal total variation image denoising, distance regularized level set evolution, and shape element for kidney segmentation from noisy ultrasound images (18). The results of this proposed method shown that the sensitivity, and specificity could reach 96% and 95% respectively. Inspired by the excellent performance of boundary detection-based kidney segmentation methods, a fully-automatic kidney image segmentation method based on CNN, which consisted of a transfer learning network, a boundary distance regression network and a kidney pixelwise classification network was developed by Yin et al. (19). Their technique used image segmentation network architecture derived from DeepLab (21) to speed up model training and improve the performance of kidney image segmentation. Moreover, image-registration data augmentation based on thin-plate splines transformation and flipping was used to train the kidney segmentation model more robust. The results showed that the proposed method had significantly better performance than other 6 state-of-the-art DL segmentation networks, namely DeepLab (21), FCNN (22), U-Net (23), SegNet (24), PSPnet (25), DeeplabV3+ (26). Chen et al. proposed a deep neural network architecture, namely Multi-branch Aware Network to segment kidney (20). The neural network mainly consists of a multi-scale feature pyramid, a multi-branch encoder and a master decoder. The design of multi-scale feature pyramid can make the network more accessible to different kinds of details at different scales. The information exchange between multi-branch encoder can reduce the loss of feature information and improve the segmentation accuracy of the network. Thus, this method not only can segment kidney images more robustly and accurately, but also can reduce the false detection rate and missed detection rate of the network.

In summary, the segmentation using DL in renal ultrasound images could not only save a considerable amount of time for radiologists and provide more objective and reliable information for the clinical work, but also contribute to the development of AI in automatic diagnosis of renal diseases. The aforementioned literature only segmented normal kidneys, not those with abnormalities, and did not set up external validators to further validate their models. Future researchers deserve a chance to solve this issue.

### 4.2 Measurement of the renal volume

Renal length is closely related to kidney function, and its change is considered as an important factor in assessing kidney status in patients with kidney disease (27). Renal bipolar length is widely used as a clinical indicator of chronicity and indirectly to estimate severity of pre-existing parenchymal damage (28). However, given that the bipolar length is a poor predictor of parenchymal volume and renal function particularly in studies utilizing MRI and CT to assess renal morphology (29). Renal volume is a relatively more reliable and accurate indicator for changes in renal length; this is confirmed by several methods for measuring renal volume, such as CT, MRI, ultrasound (30). Among the imaging technologies, ultrasound is undoubtedly a safer one for children. Kim D-W et al. aimed to develop a new automated method for renal volume measurement using hybrid learning, which integrated the DL-based U-Net model with the active contouring method based on ML to locate regions (31). They have demonstrated that the accuracy and reliability of renal volume calculation using the proposed process by comparing it with the renal volume measured by CT method. Unfortunately, the study’s sample size was insufficient, and there weren’t enough kidney samples from children in each age group to assess the model’s generalizability, statistical uncertainty, and potential bias. Overall, this is the first study on healthy children to use image pre-processing and hybrid learning to determine renal volume changes, since an age-matched normal reference value of renal volume could help in the diagnosis and prognosis of kidney diseases especially in childhood.

### 4.3 Prediction of the kidney function

Estimated glomerular filtration rate (eGFR) is the main indices for evaluating kidney function and the main basis for the diagnosis and staging of chronic kidney disease (CKD) (32). Detection of eGFR using traditional methods is invasive and non-immediate. Hence, the utilization of ultrasound for non-invasive prediction of kidney function has been proposed, as shown in **Table 2**.

**Table 2:** Summary of the application of AI in the renal ultrasound for prediction of the kidney function.

Task	Algorithms	Data source	Size	Results	Ref.
Prediction of the kidney function	Decisive area-proportional, textural features and SVM techniques	non-diabetics, non- acute renal failure, non-polycystic kidney disease, non-hydronephrosis, non-inpatient, and age between 18 to 75 years old	798 images	left-kidney: ACC=71% right-kidney: ACC=76% combining: ACC=70%	(33)
	The neural network architecture consists of 33 residual blocks as CNN-based feature extractors, and three fully connected layers of 512, 512, and 256 neurons as regressors	the view of the maximum observable kidney length	4505 images	ACC=86%	(34)
	5 ML algorithms (Nu-Support Vector Classification, C- Support Vector Classification, Random Forest, Adaptive boosting, and Xtreme gradient boosting) based on radiomics	clinical medical records of patients who underwent renal transplantation	233 patients	AUC=0.79-0.84	(35)

SVM, support vector machine; CNN, convolutional neural network; ML, machine learning; ACC, accuracy; AUC, the area under the receiver operating characteristic curve.

With texture features and SVM classification algorithm, Chen et al. designed an integrated image analysis system for diagnosing different CKD stages (33). And the accuracy of prediction results could reach 75.95%. Kuo et al. employed a deep CNN to predict eGFR based on renal ultrasound images (34). The neural network architecture they constructed was referenced from the ResNet-101 model and included 33 residual blocks and three fully connected layers consisting of 512, 512 and 256 neurons. For classifying eGFR with a threshold of 60 ml/min/1.73 m^2^, their model achieved an overall accuracy of 85.6% and area under receiver operating characteristic curve (AUC) of 0.904. Radiomics has also been discovered to be useful in evaluating kidney function. Zhu et al. developed different models based on clinical and ultrasound image features as well as radiomic features to predict the kidney function by different ML methods (35). The results of this study validated the feasibility of radiomic features in the evaluation of kidney function.

The combination of renal ultrasound imaging and AI offers the possibility of non-invasive prediction of kidney function, which can be considered as complementary evidence to aid clinical diagnosis.

### 4.4 Diagnosis of the kidney diseases

Ultrasound is widespread used in the diagnostic studies of the kidney and urinary tract. However, the diagnosis of kidney disease based on ultrasound imaging data relies on a variety of anatomical signatures, such as the renal length, the renal volume, the symmetry of the kidney, and the echogenicity of the renal parenchyma, which are usually obtained manually and exhibit a degree of interobserver dependence (36). Therefore, it is desirable to automate image analysis for a robust diagnosis of renal disease. At present, several modified AI algorithms have been developed to diagnose the various kidney diseases based on ultrasound images with the intention to help radiologists to make unbiased diagnosis, which is summarized in **Table 3**. Developing such AI algorithms helps the clinician more deeply integrate AI with renal ultrasound to find broader and practical clinical applications.

**Table 3:** Summary of the application of AI in the renal ultrasound for diagnosis of kidney disease.

Task	Algorithms	Data source	Size	Results	Ref.
Classification and diagnosis renal masses	SVM, logistic regression, naïve Bayes and quadratic discriminant analysis	clinical medical records of single center	10 AMLs; 42 RCCs	ACC=94%	(37)
	Radiomics nomogram based on ultrasound	clinical medical records of single center	600 masses	AUC=0.91 ACC=90%	(38)
	Quantitative texture information combined with tumor-to-cortex echo intensity ratio and tumor size	hyperechoic renal mass <5 cm in size	105 AMLs; 25 RCCs	AUC=0.95	(39)
	Using EffecientNet-b3 to extract features from B-mode and CEUS images, and adaptive weights are learned to fuse the features	B-mode and CEUS-mode images from two centers	9794 B-mode and CEUS-mode images	ACC=80% Sen=80% Spe=79% AUC=0.88	(40)
	An ensemble of deep neural networks (ResNet-101, ShuffleNet, and MobileNet-v2) based on transfer learning	images from available standard datasets and radiologists	4940 images	ACC=96%	(41)
Diagnosis of CAKUT	MIL combined with transfer learning	sagittal and transvers view from the first renal ultrasound scans after birth were used	86 patients with CAKUT; 96 controls	AUC=0.97 ACC=94%	(36)
	a deep MIL method based on graph convolutional networks, instance-level and bag-level supervision	clinical medical records of single center	120 CAKUT patients with 2687 images; 105 controls with 2246 images	ACC=85% Sen=86% Spe=84%	(42)
	SVM classifiers integrating texture and deep transfer learning image features	clinical medical records of single center	50 CAKUT patients; 50 controls	AUC=0.92 ACC=87%	(43)
Diagnosis and grading of hydronephrosis	An encoder-decoder framework based on U-net	coronal and transverse view of the renal ultrasound scans	Labeled dataset: 1850 images; Graded dataset: 1407 images	ACC=89%	(44)
	An Attention-Unet which consists of four convolution blocks	clinical medical records of single center	506 patients with hydronephrosis; 193 controls	Dice=0.83 Sen=90% Spe=80%	(45)
	Keras neural network consists of five convolutional layers, a fully connected layer of 400 units, and a final output layer	sagittal view of the renal ultrasound scans	2420 images	ACC=78%	(46)

CAKUT, congenital abnormalities of the kidney and urinary tract; SVM, support vector machine; CEUS, contrast-enhanced ultrasound; MIL, multi-instance learning; CNN, convolutional neural network; AML, angiomyolipoma; RCC, renal cell carcinoma ACC, accuracy; AUC, the area under the receiver operating characteristic curve; Sen, sensitivity; Spe, specificity; Dice, Dice coefficient.

#### 4.4.1 Classification and diagnosis of renal masses

Renal tumor is one of the common tumors in the urological system, and most of them are malignant. And as medical imaging technology develops by leaps and bounds in recent years, the detection rate of renal tumors is also increasing each year (47). However, only 16-19% of renal tumors are benign, especially in small renal masses (SRM, <4 cm in size) with a high benign rate of 20-30% (37, 48). Accordingly, the percutaneous renal biopsy could be considered an essential pre-treatment diagnostic procedure in clinical practice. Nevertheless, percutaneous renal biopsy has limitations, such as a high rate of false negatives, the inability to accurately diagnose SRM, and the risk of bleeding and tumor dissemination. Benign findings in the biopsy specimen will not exclude the possibility of malignancy elsewhere in the lesion (49). Therefore, accurate preoperative identification of benign and malignant renal masses can avoid unnecessary surgery and serious complications, and reduce the financial burden on patients. Among renal malignancies, renal cell carcinoma (RCC) is the most morbid and lethal pathological type, whereas angiomyolipoma (AML) is the most common type of benign renal tumor (50, 51).

As the imaging features of these two kinds of tumors not only partially overlap, but are also easily influenced by the subjective experience of radiologists, which are still challenging and controversial to identify them based on imaging features solely. Four supervised ML algorithms including quadratic discriminant analysis, logistic regression, Na Free Baye, and nonlinear- SVM were compared for accuracy in distinguishing RCC from AML, using features of tumor, cortical and medullary regions as statistical inputs. Hersh et al. demonstrated that SVM achieved the best performance in distinguishing RCC between AML with an accuracy of 94% (37). Radiomics based on ultrasound can be taken as a promising diagnostic aid, as confirmed by the studies of Li et al. and Peiman et al. (38, 39). The accuracy of the radiomics nomogram based on ultrasound constructed by Li et al. to distinguish RCC from AML was superior to the assessment performance of junior and senior radiologists (38). Peiman et al. used radiomics to extract quantitative texture information and combined tumor-to-cortex echo intensity ratio and tumor size to construct a classification model that could accurately diagnose RCC and AML with an AUC of 0.945 (39). Despite the fact that these studies have yielded promising results, they all contain limitations that can be improved, including too few images, lack of validation set, and the proportion of the number of RCCs and AMLs included significantly differs from the actual incidence in the real world. As a result, when these models are applied to the real world, the outcomes may be quite different.

Contrast-enhanced ultrasound (CEUS) is particularly indicated for differential diagnosis between solid lesions and cysts. And previous studies showed that CEUS can be used to differentiate among lesions with an equivocal enhancement at CT or MRI (52, 53). This suggests that CEUS is a promising additional diagnostic tool capable of differentiating malignant from benign renal masses. Therefore, Zhu et al. (40) constructs a multimodal ultrasound fusion network, which can independently extract features from each of the two modalities (B-mode and CEUS-mode) and learn adaptive weights to fuse features for each sample.

AI cannot merely assist in the differential diagnosis of RCC and AML, but can also precisely distinguish between multiple classes of renal abnormalities. Sudharson et al. proposed an ensemble of deep neural networks based on transfer learning, which could ideally classify renal ultrasound images into four categories, namely normal, cysts, stones, and tumors (41). Their technique combined different deep neural networks, including ResNet-101, ShuffleNet, and MobileNet-v2 for feature extraction and then used SVM for classification. The ensemble model has demonstrated better classification performance than the individual deep neural network model.

#### 4.4.2 Diagnosis of congenital abnormalities of the kidney and urinary tract

Congenital abnormalities of the kidney and urinary tract (CAKUT) are disorders caused by developmental defects of the kidney and its outflow tract, including abnormalities in the location and number of kidneys, abnormalities in the size and structure of the kidneys, and dilatation of the urinary tract, which could accelerate the progression of CKD (54). The Prevalence is estimated to be 0.04-0.6% (55). Although the widespread use of ultrasound imaging facilitates early detection of CAKUT, current methods are limited by the lack of automated processes that accurately classify diseased and normal kidneys (36).

Recent studies have introduced the contribution of their algorithms for accurate automated diagnosis of CAKUT. Yin et al. developed a clinical diagnostic model for renal ultrasound images in multiple views which built upon the transfer learning and the multi-instance learning (MIL) (36). Diagnostic performance was measured by AUC and accuracy, and was achieved to 96.5% and 93.5% respectively. The results shown that their multi-view multi-instance DL method could obtain higher classification accuracy than DL models built on individual kidney images and kidney images in one single view. Based on the graph convolutional networks to optimize the instance-level features learned by CNNs and the integrated instance-level and bag-level supervision to improve the classification, Yin et al. also developed a deep MIL method for accurately diagnosing CAKUT (42). Compared with other deep MIL methods, the performance of the proposed method could achieve the accuracy, sensitivity and specificity to 85%, 86%, 84% respectively, which demonstrated that it could improve other state-of-the-art deep MIL methods for the kidney disease diagnosis. SVM classifiers integrating texture images features and deep transfer learning image features to accurately classify the kidneys of normal children and those with CAKUT (e.g., posterior urethral valves, kidney dysplasia) were built by Zheng et al. (43). The results suggested that the proposed method performed better than classifiers based on either the transfer learning features or the conventional features alone and yielded the best classification performance for distinguishing children with CAKUT from controls.

CAKUT is a very wide range of term that encompasses a variety of congenital anomaly that may occur in the urinary tract. Different types of CAKUT can result in different sonographic findings in the urinary tract. Nevertheless, the clinical characteristics of CAKUT patients who were included in the research by Yin et al. didn’t been described in detail (36, 42). And the study by Zheng et al. only involved US images of 50 CAKUT patients and 50 controls for modeling without further validation (43). These are the points that should be taken into consideration and improved in the design of future study.

#### 4.4.3 Diagnosis and grading of hydronephrosis

Hydronephrosis is a dilatation of the kidney collecting system that occurs unilaterally or bilaterally. The grading of hydronephrosis is in accordance with the Society of Fetal Urology (SFU) study, which categorized the dilated renal pelvis, the number of calyces seen, and parenchymal atrophy into five grades of increasing severity (56). SFU grade 0 is for the normal kidney condition. Mild hydronephrosis (SFU grade 1-2) is usually considered a benign and relatively self-limiting condition that stabilizes or resolves spontaneously in most patients (57). In contrast, medium (SFU grade 3) and severe hydronephrosis (SFU grade 4) are closely related to a high risk of urinary tract infection and loss of kidney function (58, 59). Grading the severity of hydronephrosis relies on the subjective interpretation of renal ultrasound images. Hence, the emergence of AI offers a promising option for the evaluation and grading of hydronephrosis.

DL model has been used to solve hydronephrosis detecting and grading tasks in several studies. Guan et al. trained an encoder-decoder framework for ultrasonic hydronephrosis diagnosis (44). They selected U-Net to accurately segment the renal region and hydronephrosis region of the renal ultrasound images. U-Net for segmentation was trained on a labeled dataset which was annotated by professionals. And the classification network includes the encoder of U-Net for feature extraction and the Image Adaptive Classifier for image classification. Both the semantic segmentation section and the classification section shared a mutual usage of a transformation structure by separately training the encoder and decoder and loop this circle. This design can jointly utilize different supervision to automatically diagnose the severity of hydronephrosis by first segmenting and then classifying, which helps to improve the accuracy of the image classification network. A recent study by Lin et al. indicated that the Attention-Unet achieved a Dice coefficient of 0.83 for segmentation of the kidney and the dilated pelvicalyceal system (45). They further applied a fluid-to-kidney-area ratio measurement as a DL-derived biomarker for the semi-quantification of hydronephrosis. This DL method has been confirmed to provide an objective evaluation of pediatric hydronephrosis. A five layers CNN model developed from the Keras neural network API with Tensorflow to grade hydronephrosis ultrasound images was conducted by Smail et al. (46). The CNN model successfully achieved an average accuracy of 78% when classifying SFU grades 1-2 vs. SFU grades 3-4.

The current studies have explored the application of DL for detection and grading of hydronephrosis, validating the potential of the DL method to be capable of serving as a decision aid for clinical practice. However, the aforementioned study was retrospective, so it was not balanced in the types of SFU patients that included. A dataset with a balanced type and larger amount of data to construct a DL model may result in better performance and more accurately assist physicians in diagnosing hydronephrosis.

## 5 Challenges and prospects

Despite the unprecedented and rapid development of AI technology in medical imaging, it is still far from being applied to various hospitals on a large scale. At present, there are many limitations to the various studies conducted on the application of AI to renal ultrasound. Controversies in radiomics applications include how to standardize ultrasound images, manual segmentation of images is both time-consuming and unstable, and unbalanced datasets used to construct models can lead to overfitting. On the other hand, in DL applications, the lack of large-scale public datasets, high requirements for image quality, and ultrasound devices from different manufacturers and heterogeneity of operators may lead to the variability in the training process. In addition, when there are more restrictions on the inclusion and exclusion criteria of the data, data censoring and bias are serious, which can also lead to AI models that deviate from the real world and have low generalization capabilities.

At the meantime, these also offer a new impetus for the development of renal ultrasound and show the broad prospect of AI-powered ultrasound in the future. Prospective multicenter studies are now urgently necessary, so how to eliminate discrepancies between different ultrasound devices and imaging parameters is a primary problem to be solved. The heterogeneity and variability of ultrasound data acquisition methods are the main obstacles that limit the comparison and generalization of different methods across different tasks. Therefore, the establishment of a standardized database for ultrasound applications is one of the directions for further research in the future. And the necessity of more robust methods to deal with speckle noise in ultrasound images and to automate the segmentation process (16).

In the subfield of renal ultrasound, there are still many applications that deserve in-depth study. For instance, in the treatment of renal masses, AI enables an optimized surgical approach known as robot-assisted partial nephrectomy. Robot-assisted partial nephrectomy has shown a significant improvement in the preservation of renal function, with no significant difference from radical nephrectomy in terms of overall survival, cancer-specific survival, recurrence or comorbidities (60, 61). However, one of the problems of AI in the diagnosis and treatment of kidney disease is how to reliably evaluate the remaining functional part of the kidney and choose the right surgical scope appropriately. Therefore, in addition to the diagnosis of benign and malignant renal tumors, the proper estimation of the remaining portion of the kidney for accurate surgical scope should be addressed when using AI for renal ultrasound.

## 6 Conclusion

AI, particularly DL, is progressively changing the field of medical imaging, giving rise to improved performance in the kidney segmentation, prediction of the kidney function, diagnosis of the kidney diseases. Due to its powerful image processing capability, fast computing speed and fatigue-free, AI applied to ultrasound is becoming more mature and coming closer to routine clinical applications (62). In the future, AI technology would gradually become inseparable from clinical work. However, we cannot directly apply various algorithms to clinical tasks. Given to the merits and weakness of varied algorithms, we need to choose the algorithm carefully to make the best use of its advantages according to the specific ultrasound task. With the rapid development of AI technology, clinicians must quickly adapt to their new roles as technology users and patient advocates, and refine their expertise for the benefit of their patients (63).

## Author contributions

X-WC and Y-XP established the design and conception of the paper; TX, X-YZ, and NY explored the literature data; TX provided the first draft of the manuscript, which was discussed and revised critically for intellectual content by X-YZ, NY, X-FP, G-QC, X-WC, and Y-XP. All authors contributed to the article and approved the submitted version.
